# Synergistic Effects of Polypyrrole Nanotubes and Magnetite Nanoparticles in PEO-Based Composites for Solid Polymer Electrolyte

**DOI:** 10.1021/acsami.6c01988

**Published:** 2026-06-09

**Authors:** Andrei Munteanu, Marek Jurca, Lenka Munteanu, Michal Sedlacik, Constantin Bubulinca

**Affiliations:** Centre of Polymer Systems, University Institute, 48362 Tomas Bata University in Zlín, Trida T. Bati 5678, Zlín 760 01, Czech Republic

**Keywords:** magnetic composite, poly(ethylene oxide), solid-state battery, solid polymer electrolyte, polypyrrole nanotubes, sodium carboxymethyl cellulose

## Abstract

A showcase study in which magnetic particles are investigated as a filler, mainly for the development of solid polymer electrolytes, is presented. The magnetic particles, in the form of polypyrrole (PPy) nanotubes, were decorated with different amounts of magnetite nanoparticles, which can be tuned through synthesis. The particles were dispersed in high molecular weight poly­(ethylene oxide) blended with sodium carboxymethyl cellulose, preparing pellets and thin films. An external magnetic field was used to obtain anisotropic thin films, with the differences described in detail. After solid characterization, we show that, depending on the synthesis of composite particles and the fabrication methods of the pellets and films, we can alter various properties—magnetic (*M*_sat_ ∼ 30–60 emu/g), electric (σ_ionic_ ∼ 10^–7^–10^–4^ S/cm), and mechanical (G’ ∼ 8–10 MPa). The presence of the filler improved the shear modulus (∼60 MPa), competing with dendrite propagation while offering improved thermal stability at elevated temperatures (80°C). Depending on the preparation and composition of the filler material, the ionic conductivity always improved with the presence of the PPy/Fe_3_O_4_, with a specific sample reaching ∼10^–4^ S/cm at room temperature. The present work is a new strategy to improve the properties of solid polymer electrolytes and can pave the way for more efficient and more competitive magnetic composites used for energy storage.

## Graphical abstract

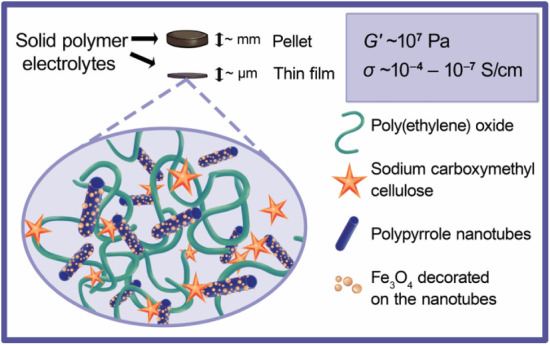

## Introduction

The enforcement of the “Green Deal” and the need to shift energy production toward more environmental friendly (i.e., pollution-free) methods have revolutionized energy storage.^1^ Many of the current electricity generation systems (photovoltaic systems, wind farms, etc.) produce more energy than can be effectively consumed at any given time, which reduces the overall efficiency.^2^ It is clear that the main challenge is to improve the energy storage options before optimizing green energy harvesting. Current batteries work through either liquid or solid-state electrolytes (SSEs). During the charge and discharge cycles, needle-like structures, commonly known as dendrites,^3^ grow from the anode, causing problems ranging from performance issues to battery destruction, with explosions capable of seriously damaging industrial complexes being reported.^4^ The dendrite formation is related to the viscosity of the electrolyte; thus, SSEs can suppress the dendrite formation.^5^ It is widely accepted that an SSE can only be used in commercial batteries if the shear modulus is at least two–three times that of metallic lithium and conductivity exceeds 10^–4^ S/cm, respectively.^6^ However, SSEs also have a disadvantage, namely significantly lower ionic conductivity, the improvement of which is the most researched topic in this field.^7^

Polymer-based SSEs are a promising candidate as the ionic conductivity is mainly driven by the segmental dynamics of the polymers, which carry the ions or enable ion hopping depending on the molecular weight of the polymer.^8,9^ This type of SSE can achieve very high ionic conductivities, especially if the molecular weight is low.^10^ Poly­(ethylene oxide) (PEO) is among the most common and serious candidates.^11^ Apart from ionic conductivity, it possesses various unique properties and has been widely used for decades in various applications.^12–15^ However, to obtain competitive values of ionic conductivity for a simple linear PEO-based electrolyte, a low molecular weight is required, thus making it soft enough to allow dendrite formation.^16^ Thankfully, due to the vast research on different applications, numerous PEOs with diverse morphologies and their derivatives exist to investigate as potential electrolytes.^17,18^ The pliable nature of PEO allows for easy and cheap access to its derivatives,^19^ with the fabrication conditions being scalable. Various sophisticated solutions were already proposed. To name a few, Glynos et al. took advantage of the good mechanical properties of polystyrene (PS) and prepared PEO–PS miktoarm stars, which were embedded in a PEO-based electrolyte and achieved sufficient mechanical strength with conductivity in the order of 10^–5^–10^–4^ S/cm at room temperature.^20^ In another work, this group showed that integrating poly­(methyl methacrylate) (PMMA)-based highly functional star-shaped nanoparticles enhanced both the mechanical properties and ionic conductivity by one and two orders of magnitude, respectively, in comparison to linear PMMA blends.^21^ Other approaches include the incorporation of fillers^22,23^ or utilizing gels^24^ which can be utilized for the liquid counterparts. Lastly, creating 3D networks within the electrolyte is another emerging proposition to inhibit the propagation of dendrites.^25^

A different approach involves magnetic materials with oriented pathways inside the solid electrolyte, which improve the ion kinetics during the charging/discharging process by adjusting the magnetic field, magnetic particle loading, and acid etching time.^26^ Thus, magnetic materials have been dispersed in ionic liquids to create solid electrolytes, and their excellent conductivity and mechanical properties were investigated with fluctuating magnetic fields.^27^

Magnetic composites were recently reported for a ceramic-based SSE.^28^ Regarding polymer materials, magnetorheological elastomers (MREs) could potentially work as either a polymer-based SSE or a separator. Magnetorheological elastomers are composed of soft magnetic particles and a nonmagnetic polymeric matrix.^29,30^ Unlike other fillers, magnetic particles can be permanently aligned during sample preparation, forming an anisotropic material.^31^ Poly­(ethylene oxide), being soluble in many common solvents, including water,^32^ is a great candidate as the medium for these MREs. Anisotropic MREs have higher stiffness without compromising their other properties.^33^ The interparticle distances within anisotropic MREs are smaller, which also increases the overall conductivity through ion hopping.^34^ Very few cases of SSEs with magnetic properties exist in the literature, including a PEO-based MRE; however, the reported ionic conductivity is several factors below the minimum commercial values.^35^ Aji et al. managed to prepare an MRE-based electrolyte utilizing poly­(vinyl alcohol) (PVA) and iron oxide nanoparticles, reaching the targeted ionic conductivity.^36^ Several works have been reported on agarose-magnetite-based electrolytes, achieving the alignment of the polymer and enabling these materials to be used in other applications.^37,38^ However, the mechanical properties of the latter two systems were not competitive. Regarding SSEs, to our knowledge, no magnetic SSE batteries have been reported, although there are a handful of MRE-based liquid electrolytes that have both improved the overall properties of batteries and addressed specific issues.^39,40^

In this work, we present a strategy to develop and prepare SSEs from composites based on PEO and polypyrrole (PPy) nanotubes decorated with magnetite nanoparticles. Poly­(ethylene oxide) was used due to the above-mentioned advantages. Polypyrrole, on the other hand, is a material used in electrolytes since 80s, and the ion-exchanging mechanisms are generally understood; however, it is rarely in the spotlight.^41,42^ In our case, the composite PPy/Fe_3_O_4_ particles were mainly used as a magnetic filler. The tube-like morphology of the PPy was selected as it has not been investigated before and also because of the overall improvements that nanotube fillers can provide in batteries.^43^ Composites, both in bulk form and thin films, are investigated. Thin-film SSEs, specifically, were prepared in the presence and absence of a magnetic field to study the influence of PPy/Fe_3_O_4_ orientation (anisotropic vs isotropic ordering of the filler) on the conductive properties. Lastly, other important aspects of SSEs, such as the affinity between components and thermal stability, which are usually neglected, were described.

## Materials and Methods

### Materials

For the preparation of the battery components, three main components were utilized. Sodium carboxymethyl cellulose (Na-CMC) with *M*_w_ ∼ 250 000 g/mol, provided by Sigma-Aldrich (USA), was selected to improve ionic conductivity due to its widespread use in thin films.^44–46^ Poly­(ethylene oxide) with a high *M*_w_ ∼ 200 000 g/mol, also provided by Sigma-Aldrich, was utilized for its good mechanical properties and affinity with PEO-based electrolytes. Lastly, PPy nanotubes with various contents of magnetite nanoparticles were synthesized in this work. These nanotubes are magnetic, a property essential for preparing materials with anisotropic structures. All three components were received/produced in powder form.

### Synthesis of the PPy/Fe_3_O_4_ Nanotubes

The electric and magnetic properties of the PPy nanotubes can be easily tuned through synthesis by controlling their structure/morphology via different synthetic pathways.^47,48^ However, when preparing the PPy nanotubes, globular PPy is also obtained, which is generally neglected. To diminish the PPy globular particles, we further optimized the synthesis of these nanotubes. Key differences between the established synthesis and the current method include the modification of the concentration of ammonium hydroxide^49^ and the amount of methyl orange used, together with the mixing conditions. In detail, the synthesis of the magnetic PPy nanotubes was performed in two steps: pyrrole polymerization followed by the precipitation of magnetite nanoparticles. Two types of PPy nanotubes were synthesized using different molarities of iron­(III) chloride hexahydrate (FeCl_3_·6H_2_O) (>99% purity). The amount of each component used in the reaction is noted in Table 1 with the corresponding mole ratio of FeCl_3_·6H_2_O over pyrrole represented as *n*. The ratios *n* = 2.5 and *n* = 6 were selected based on previous works, with the former yielding the most conductive nanotubes and the latter the most magnetic.^47,48^ In the first step of the synthesis, the pyrrole monomer (>97% purity, 0.2 M) and methyl orange dye (0.016 M, >99% purity) were dissolved in 100 mL of water and were thoroughly mixed. The dye acts as a template for the growth of the tube-like morphology; without it, globular PPy would result instead.^50^ In the meantime, a second solution was prepared by dissolving FeCl_3_·6H_2_O in water (0.5 M in 100 mL and 1.2 M in 100 mL for *n* = 2.5 and for *n* = 6, respectively). Under continuous stirring, the iron chloride solution was slowly added to the monomer solution, and the reaction was allowed to proceed until the complete polymerization of the pyrrole was achieved (30 min), resulting in the formation of PPy nanotubes, indicated by the solution becoming a mixture that notably changed color to dark brown and increased in viscosity. For *n* = 2.5, most of the FeCl_3_ is consumed during pyrrole polymerization, resulting in the formation of PPy nanotubes in the base form. For *n* = 6, the excess of unreacted Fe^3+^ leads to the generation of a Fe^3+^/Fe^2+^ molar ratio of approximately 2:1, which is stoichiometric for magnetite formation, thereby decorating the PPy nanotubes with magnetite nanoparticles. The final molar concentrations of the reaction mixture are 0.1 M of pyrrole, 0.008 M of methyl orange, and 0.25 and 0.6 M of FeCl_3_ for *n* = 2.5 and *n* = 6, respectively. The decorating of the PPy nanotubes with magnetite nanoparticles, as the second step of the synthesis, was performed as follows: an excessive amount of ammonium hydroxide (4 M) was slowly added to the resulting PPy mixture while constantly measuring the pH using a pH meter. Once the mixture’s pH reached 10, the procedure was stopped. Lastly, the coated PPy nanotubes were filtered and washed with ethanol, resulting in a wet dark brown powder that was dried overnight at 60 °C. The nanotubes with *n* = 2.5, named PPy2.5, are more conductive, slightly magnetic, with a higher aspect ratio. The nanotubes with *n* = 6, named PPy6, on the other hand, are less conductive with a smaller aspect ratio, in exchange for superior magnetic properties, with visual illustrations shown in Figure 1. The encircled images indicate the PPy6 and PPy2.5 powders that are able to hold the weight of a Petri dish using a magnet. All chemicals used were obtained from Sigma-Aldrich. All steps were performed under constant stirring (400 rpm) at laboratory temperature. It is important to note that previously established procedures yield globular PPy impurities; however, this method results in highly pure PPy nanotubes.^47,48^

**Table 1:** Reactants Used for the Synthesis of PPy/Fe_3_O_4_ Nanotubes

Code name	*n*	pyrrole (mL)	FeCl_3_·6H_2_O (g)	water (mL)	methyl orange (mg)
PPy2.5	2.5	1.4	13.52	200	520
PPy6	6	1.4	32.45	200	520

**Figure 1:**
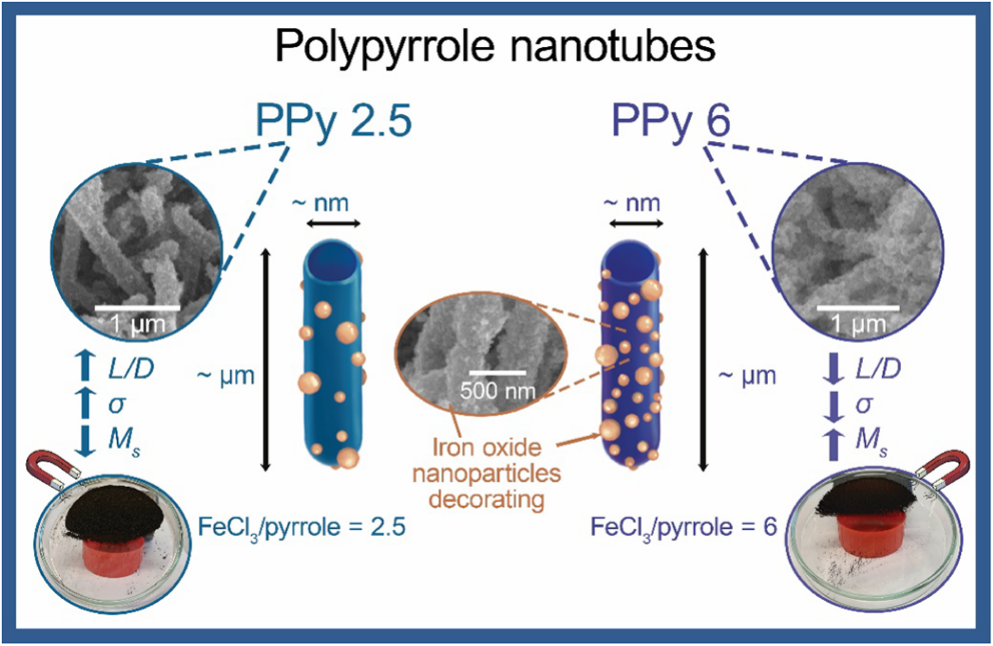
Visual illustration of PPy2.5 and PPy6 nanotubes highlighting their differences. The encircled micrographs show images from scanning electron microscopy. The encircled pictures highlight that PPy6 is more responsive to the magnetic field.

### Sample Preparation

#### Initial Powder

The preparation of the pellets and thin films is illustrated and briefly described in Figure 2. Pure PEO samples, further noted as “P-PEO,” were prepared by grinding the polymeric powder in a mortar with a pestle by hand for about 20 min. Similarly, a blend of PEO and Na-CMC, further noted as “PEO-M,” was prepared by grinding the two components in a 9:1 (PEO to Na-CMC mass ratio) also for 20 min. A common phenomenon among PPy particles is their agglomeration;^51^ therefore, before preparation, the magnetic PPy nanotubes were extensively ground in a mortar for 30 min. Then, the corresponding amounts of PPy nanotubes and PEO-M were mixed and ground in the same mortar for an additional 20 min. In Table 2 below, the weight fractions for all of the components are presented. All samples are isotropic, with the first number of the codename indicating the wt % of the corresponding nanotube type, while future anisotropic samples will be marked with the initial “A” in front. For instance, the 5-PPy2.5 sample contains 95 wt % of PEO-M and 5 wt % of PPy2.5 nanotubes. The filler mass fractions of 5% and 10% were considered, as in the regime above 10%, the ionic conductivity of particle-filled electrolytes usually decreases after reaching a maximum.^23^ Using these powders, two types of samples were prepared: disks and thin films.

**Figure 2:**
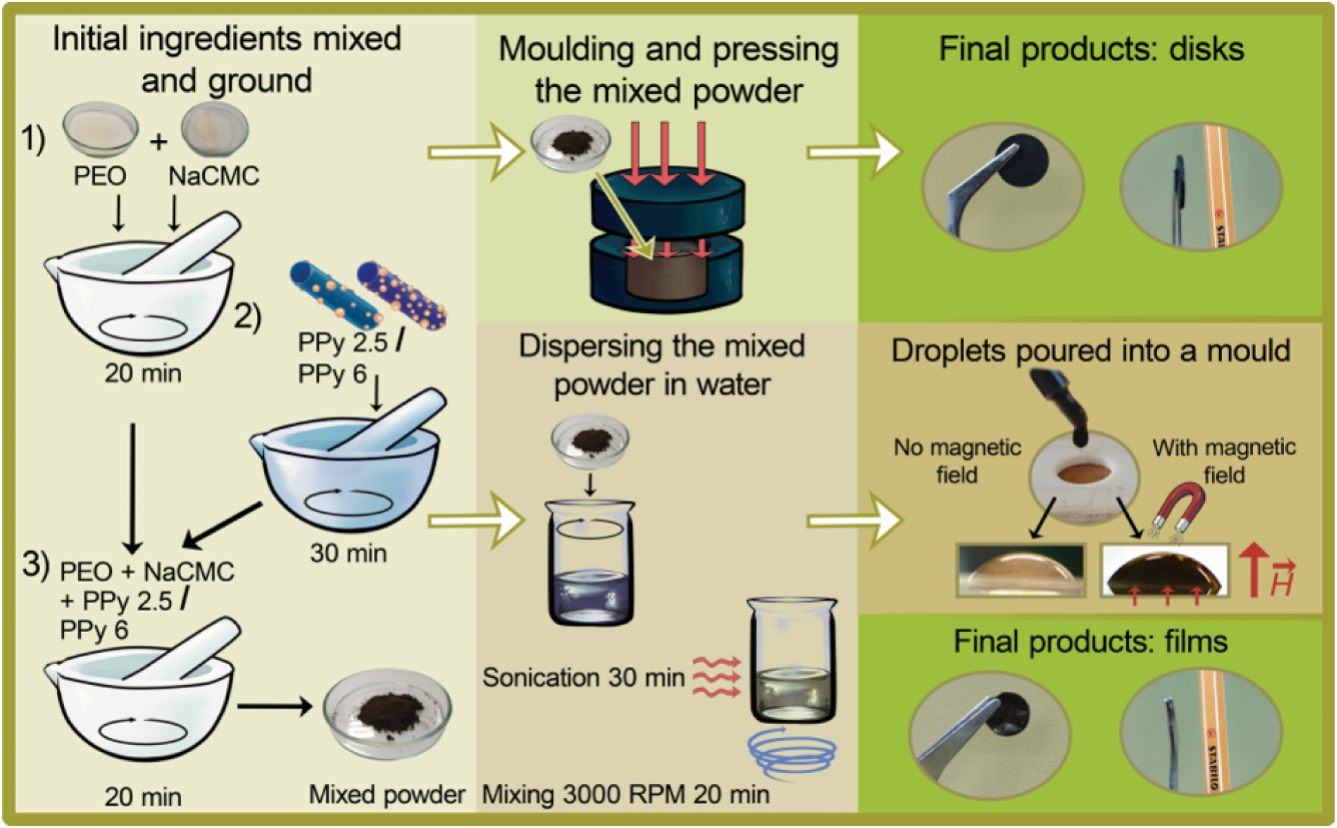
Step-by-step fabrication process of pellets and thin films. The PEO, Na-CMC, and the corresponding PPy nanotubes were mixed through extensive grinding (left part). The resulting powder was compressed through a hydraulic press, resulting in the pellet disk (top part). For the thin films (bottom part), the powder was dissolved in water (20 g/L), sonicated for 30 min, and mechanically stirred for 20 min. Then, the solution was cast in Teflon molds with or without the presence of a magnetic field. The encircled images highlight the final product and compare their thickness with a pen.

**Table 2:** Weight Fractions for Ppy-Based Powders^a^

Materials	5-PPy2.5	10-PPy2.5	5-PPy6	10-PPy6
PEO-M	0.95	0.9	0.95	0.9
PPy2.5	0.05	0.1	—	—
PPy6	—	—	0.05	0.1

a The same fractions were used for the corresponding anisotropic samples.

#### Preparation of Disks

The corresponding powder was left overnight in a static vacuum and then inserted into a disk-shaped mold (R = 13 mm). Samples of various thicknesses (1–2 mm) were obtained through a hydraulic press, Redats H-370, at 53 MPa pressure for 30 s. Only isotropic disks were prepared.

#### Preparation of Thin Films

The powder was left in a static vacuum overnight and then dispersed in water at a ratio of 200 mg/10 mL. The solution was then sonicated for 30 min and mixed in a vortex mixer at 3000 rpm for 15 min until the PEO-M was completely dissolved and the PPy tubes were well dispersed without any coagulates. Right after mixing, a part of the dispersion was injected into a thin Teflon mold (R = 5 and 8 mm) and was left to dry. In this way, isotropic thin films were produced. Anisotropic films were produced with the same process, with the addition of a magnetic field using neodymium magnets. The magnetic induction inside the mold was 0.15 ± 0.02 T, measured through the FH 51 T-meter (MAGNET-PHYSIK, Germany). Due to the superior magnetic properties of the PPy6 tubes, only the corresponding films were fabricated.

## Characterization

### Morpho-Structural Characterization

The characteristic morphology of the polymer composite with magnetic particles, in the form of thin films and pellets, was investigated by a NanoSEM 450 (FEI) field emission scanning electron microscope (FESEM) equipped with a TEAM EDS analytical system. The FESEM system shows high versatility in operating modes, including high- and low-vacuum.

An X-ray diffraction (XRD) analysis was performed with a Rigaku Mini-Flex 600 desktop diffractometer employing Cu Kα radiation, scanning from 3° to 90° (2θ) at a scan rate of 5°/min. Prior to testing, samples in powder form were dried after being suspended in ethanol and sonicated for 10 min using an ultrasonic homogenizer apparatus (Dr. Hielscher GmbH, Germany) equipped with the ultrasonic horn S7.

### Electrochemical Characterization

The evaluation of ionic conductivity was supported by electrochemical impedance, which gives the volume resistivity value used in the calculation formula:

1 $$\sigma_{i}=\frac{T}{R_{b}\cdot A}$$

where σ_i_ is ionic conductivity (S/cm), *T* is thickness of the sample (thin film or pellet), *R*_*b*_ is bulk resistance, and *A* is cross-sectional area.

A Swagelok cell was used to evaluate electrochemical impedance spectra. The cells were assembled in air and tested at room temperature and at 60 °C. The electrochemical assessment was performed on an Autolab PGSTAT-128 N Potentiostat and a Bio-Logic BCS-810 multichannel battery tester. Moreover, we evaluated the ionic conductivity error based on propagating the uncertainties from eq 1, combining errors from the sample’s thickness, area, and bulk resistance. The relative error $\left(\frac{\Delta \sigma }{\sigma }\right)$ can be estimated using the following formula:

2 $$\frac{\Delta \sigma }{\sigma }=\sqrt{{\left(\frac{\Delta L}{L}\right)}^{2}+{\left(\frac{\Delta A}{A}\right)}^{2}+{\left(\frac{\Delta R_{b}}{R_{b}}\right)}^{2}}$$

where Δ*L*, Δ*A,* and Δ*R_b_* are the absolute errors of the respective measurements.

### Rheometry

The mechanical properties of the disks were investigated using the Physica MCR 502 rheometer (Anton Paar, Austria). The device was equipped with the P-PTD200/62/TG Peltier and the H-PTD200 hood. The specimens were cut into 10 mm diameter disks using a hydraulic press and were investigated using the corresponding parallel plate geometry. Due to their high stiffness, before testing, each sample was trimmed using sandpaper to roughen its surface and suppress slippage. For the same reason, at room temperature, the specimens were tested with a normal force of 1.5 N applied. The samples were investigated mainly through dynamic frequency within the linear regime, with the strain being 0.005% and 0.1% at 25 and 80 °C (no normal force applied), while a mild nitrogen flow (300 mL/h) was utilized. Before each frequency sweep, the samples were left under small amplitude oscillatory shear until equilibrium was reached; thus, all samples were relatively stable at elevated temperatures.

### Vibrating Sample Magnetometry

The magnetic properties of the rods were investigated by evaluating magnetic hysteresis curves using a vibrating sample magnetometer, Model 7407 (Lakeshore, USA). The samples were swept from −10 kOe to 10 kOe at room temperature. The anisotropic films were measured with the structures being perpendicular to the magnetic field. To calculate the saturation magnetization, the hysteresis curves were fit using the following approximation based on the Fröhlich-Kennelly model: $M=\frac{H}{\alpha +\beta \left|H\right|}$ , with *M* and *H* being the measured mass-specific magnetization and the applied magnetic field, respectively, and α and β being fitting parameters. The saturation magnetization, *M*_s_, was taken as the $\underset{H\to \infty }{\lim}M\left(H\right)$ . The susceptibility was calculated for all samples through fitting in the linear region between −250 and 250 Oe.

## Results and Discussion

### Morpho-Structural Characteristics of the Samples

The specific morphological structure of the PPy/Fe_3_O_4_ magnetic particles and the PEO-based composite material containing PPy/Fe_3_O_4_ filler is shown in Figure 3. At different stoichiometric ratios (PPy2.5 and PPy6), well-dispersed PPy/Fe_3_O_4_ nanotubes are observed without contamination or granular PPy, confirming the improved synthesis method. Furthermore, the magnetite nanoparticles uniformly coat the entire surface of the PPy nanotubes, with a notably higher coverage at *n* = 6, attributed to the increased magnetite concentration (see Figure 3, 10 min mix).

**Figure 3:**
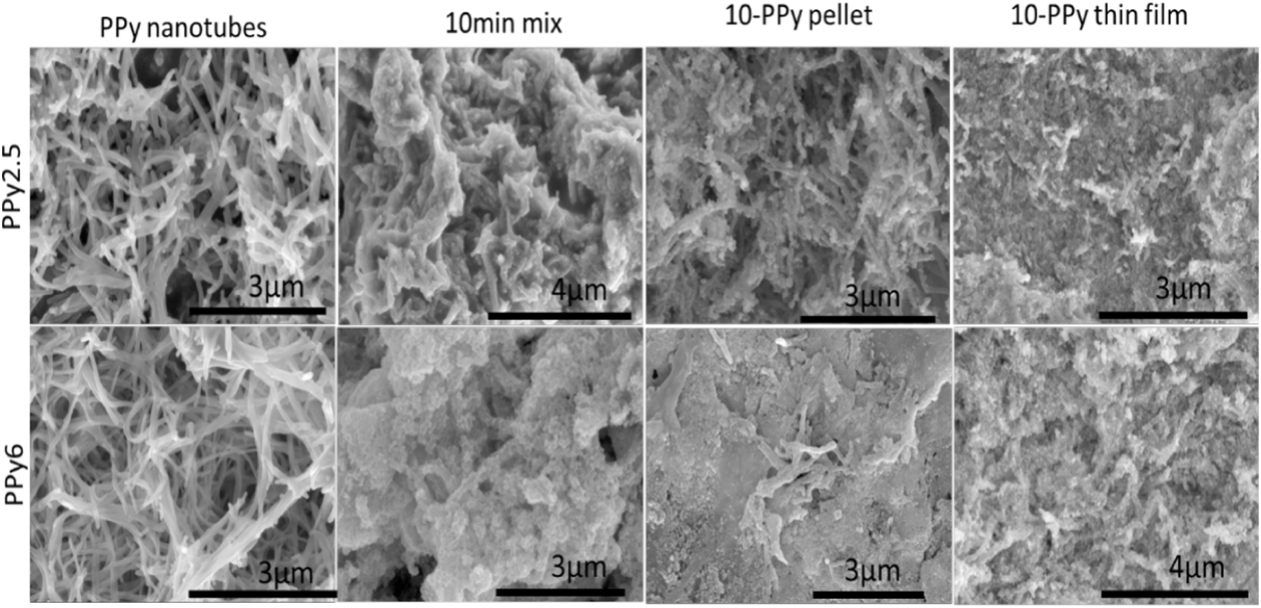
SEM of the PPy nanotubes decorated with magnetite and their corresponding PEO composites.

The specific structure of the composite, based on PEO and PPy/Fe_3_O_4_ (with a mass ratio of 9/1), was further investigated through the preparation of pellets and thin films. The surface morphology of the pellet form reveals a tubular architecture decorated with PPy nanotubes and PEO particles for *n* = 2.5, while the sample with a higher molar content of magnetite *(n* = 6) shows incorporation into a PEO matrix. A similar structural morphological pattern is observed for composites in the form of thin films. However, in this configuration, the dispersion of PPy nanotubes, entangled and decorated with magnetite within the PEO matrix, is more distinctively highlighted. Consistent with the pellet morphology, the thin-film structure remains comparable across both molar ratios, with a noticeable excess of magnetite for *n* = 6.

To identify possible impurities that may arise during the synthesis process, XRD analysis was performed (Figure 4). Two samples containing PPy/Fe_3_O_4_ components with different stoichiometric concentrations were investigated. The diffraction patterns in Figure 4 reveal the presence of a broad peak at 2θ < 20°, which is characteristic of amorphous PPy.^47^ This broad peak originates from the scattering of PPy chains at the interplanar spacing.^47^

**Figure 4:**
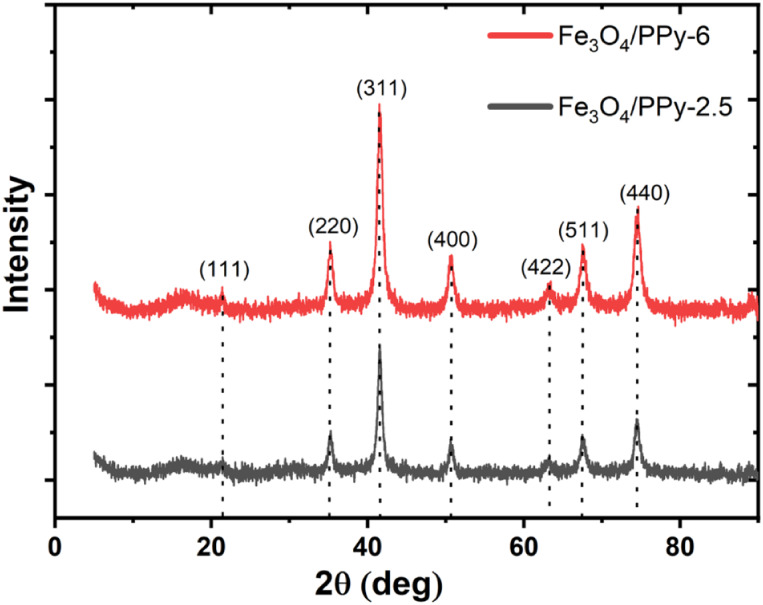
X-ray diffraction pattern of PPy/Fe_3_O_4_ composite nanotubes prepared at PPy2.5 and PPy6.

The diffraction patterns for both samples exhibit similar peaks at 2θ = 22°, 36°, 41°, 50.5°, 63°, 68°, and 76°, corresponding to the crystallographic planes 111, 220, 311, 400, 422, 511, and 440, respectively. These peaks confirm the crystalline structure of magnetite. No additional peaks were detected in the diffractograms, indicating the absence of secondary derivative structures. Furthermore, an increase in peak intensity and relative peak area was observed for PPy6, which correlates with the growth of the crystalline phase as the magnetite concentration increases.

### Ionic Conductivity

Ionic conductivity is a key parameter for SSEs, as it determines how efficiently ions can migrate between electrodes. To evaluate this property, we investigated the synergistic effect of PPy and magnetite to assess the potential of PEO-based composite materials containing PPy and magnetite as SSEs. Electrochemical impedance spectroscopy was performed on both isotropic and anisotropic samples, prepared in the form of pellets and thin films, at room temperature and at 60 °C. By extracting the bulk resistance values from the Nyquist plots and combining these with measurements of sample thickness and electrode area, the ionic conductivity and the corresponding error were calculated according to eqs 1 and 2. The resulting values are summarized in Tables 3 and 4.

**Table 3:** Ionic Conductivity of Thin Films

	σ (S/cm)		
Material (thin film)	22 °C	60 °C	Thickness (μm)
10-PPy2.5	(7.00 ± 0.8) × 10^–7^	(8.55 ± 0.8) × 10^–7^	70
A10-PPy2.5	(1.59 ± 0.3) × 10^–6^	(6.80 ± 0.3) × 10^–6^	90
10-PPy6	(1.40 ± 0.2) × 10^–7^	(4.42 ± 0.13) × 10^–7^	113
A10-PPy6	(4.15 ± 0.4) × 10^–7^	(7.21 ± 0.42) × 10^–7^	50
P-PEO	(7.53 ± 1.2) × 10^–8^	(5.95 ± 1.3) × 10^–7^	50

**Table 4:** Ionic Conductivity of Pellets

	σ (S/cm)		
Material (pellet)	22 °C	60 °C	Thickness (mm)
P-PEO	(1.04±0.32) × 10^–9^	(1.59 + 0.48) × 10^–8^	1.47
PEO-M	(3.58±0.10) × 10^–6^	(2.86±0.12) × 10^–4^	1.21
5-PPy2.5	(3.65±0.28) × 10^–5^	(5.27±0.4) × 10^–5^	1.50
10-PPy2.5	(1.06±0.08) × 10^–4^	(3.06±0.11) × 10^–4^	1.70
10-PPy6	(3.07±0.23) × 10^–6^	(6.40±0.19) × 10^–6^	1.55

For the isotropic composite materials in the form of pellets, the ionic conductivity of pure PEO at room temperature and 60 °C shows only slight variation. This behavior is attributed to the increased amorphous region and enhanced segmental motion of polymer chains, which facilitate ion transfer at elevated temperatures.^44^ A similar trend is observed for the thin films, where the ionic conductivity is slightly higher, reaching (5.95 ± 1.3) × 10^–7^ S/cm at 60 °C.

The incorporation of Na-CMC into the PEO matrix significantly improves ionic conductivity, achieving (2.86 ± 0.12) × 10^–4^ S/cm at higher temperatures (pellet). This enhancement is due to the high density of −COO^–^ and −OH groups, which act as complexation sites, reduce crystallinity,^52^ and promote ionic transport. Further improvement is observed upon introducing PPy2.5 into the PEO matrix, with ionic conductivity reaching (3.06 ± 0.11) × 10^–4^ S/cm for the 10-PPy2.5 pellet sample at 60 °C. Our results are comparable with solvent-free PEO-based SPE,^53^ PEO–PS miktoarm stars,^20^ and polymer electrolytes with ceramic nanowire fillers.^54^ However, the composite solid polymer electrolyte with PPy6 exhibits lower ionic conductivity ((6.40 ± 0.19) × 10^–6^ S/cm) under similar testing conditions. This decrease can be attributed to filler agglomeration caused by the higher magnetite concentration, which leads to the formation of larger particles and consequently reduces overall polymer chain mobility.

However, thin-film samples exhibit inferior ionic conductivity compared to pellets, likely due to differences in the dispersion and stability of the amorphous phase, as well as improved ionic pathways arising from compaction at grain boundaries in pellet form. To illustrate these findings, Nyquist plots (Figure 5) are presented, comparing the impedance behavior of the various polymer composites. The plots display the characteristic semicircular arcs associated with the charge transfer resistance and capacitive behavior.

**Figure 5:**
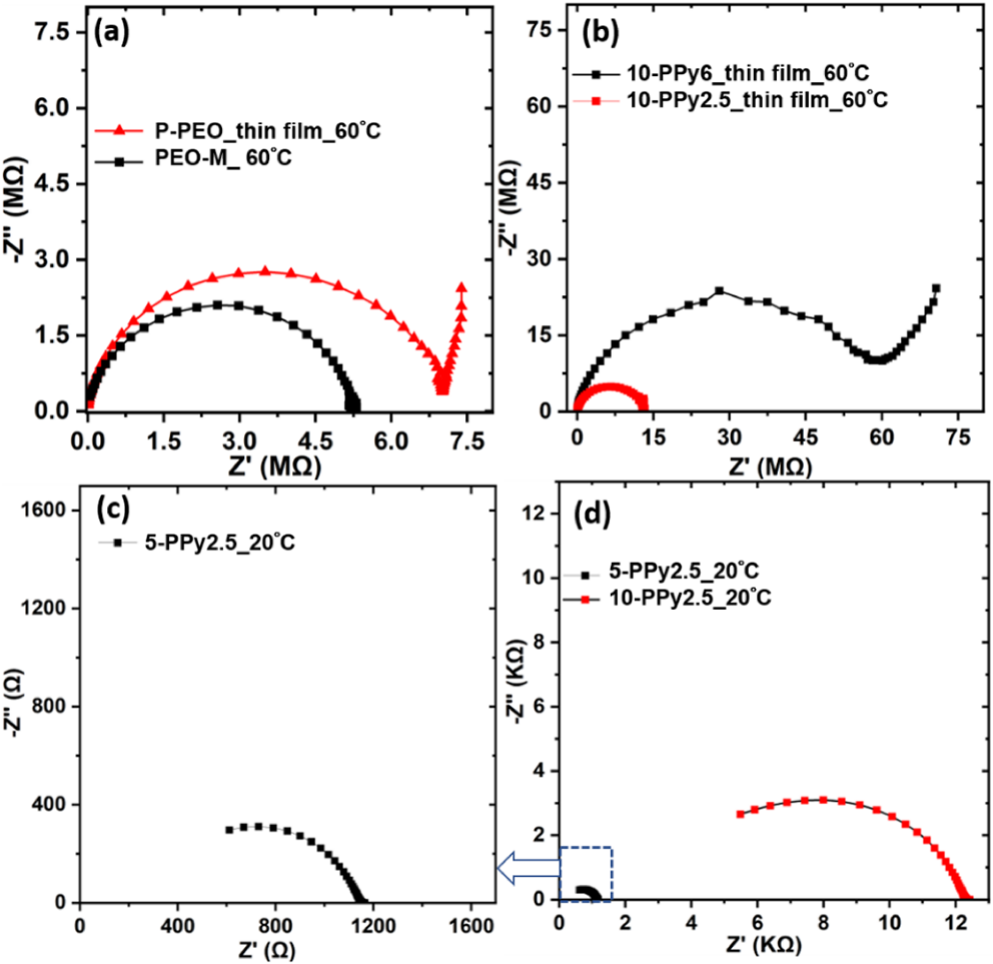
Specific Nyquist impedance diagrams of pure PEO and PEO–PPy/Fe_3_O_4_ composites with *n* = 6 (a, b), *n* = 2.5 (b, d), and magnified 5-PPy2.5 (c) at room temperature and 60 °C in the form of pellets and thin films.

Interfacial resistance varies significantly, ranging from 1 kΩ for isotropic 10-PPy2.5 pellets at room temperature to 60 MΩ for 10-PPy6 thin films at 60 °C. Overall, the magnetic composite materials based on PEO-M, magnetite nanoparticles, and PPy nanotubes demonstrate promising ionic conductivity at room temperature, making them strong candidates for solid polymer electrolytes.

### Magnetic Properties

The magnetic hysteresis loops are presented in Figure 6 for the most relevant samples. All samples achieve magnetizations close to saturation at relatively low fields, which is an attractive feature for applications. The *M*_s_ values for each sample are presented in Table 5. Regarding the PPy/Fe_3_O_4_ nanotubes, considering them to be only partly composed of magnetite, the *M*_s_ is extremely high, especially for PPy6, considering that various pure Fe_3_O_4_ nanoparticles are usually reported with similar *M*_s_ values. In comparison to previous works,^47,48^ the *M*_s_ was improved for both types of PPy nanotubes thanks to the modifications during synthesis.

**Figure 6:**
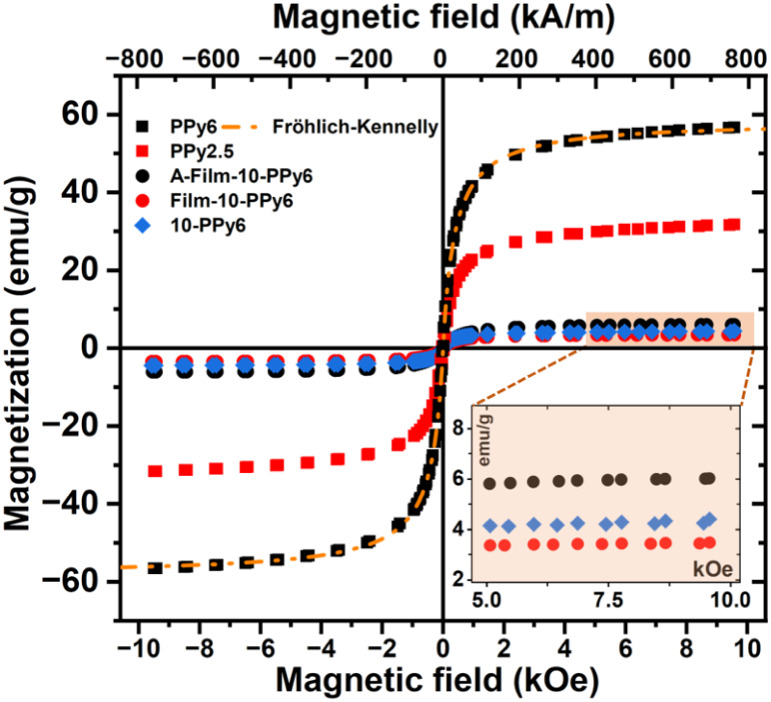
Magnetic curves for PPy6 and PPy2.5 nanotubes (black and red squares), anisotropic and isotropic films (black and red circles), and the 10-PPy6 mixture for the preparation of the films. The dotted line corresponds to a Fröhlich-Kennelly approximation for the PPy6 nanotubes. The inset magnifies the curve in the neighborhood of the films and the 10-PPy6 mixture.

**Table 5:** Various Magnetic Properties Extracted from the Hysteresis Curves for the PPy Nanotubes 10-PPy6 and the SSE Films

	*M*_r_ (emu/g)	*H*_ci_ (Oe)	*M*_s_ (emu/g)	χ_m_ (cm^3^/g)	*H*_EA_ (Oe)
PPy2.5	2.24	32.2	32.47	0.053	0.9
PPy6	1.42	11.0	58.31	1.026	0.5
A-Film-10-PPy6	0.09	7.8	6.42	0.009	3.9
Film-10-PPy6	0.53	6.3	3.65	0.007	3.3
Powder-10-PPy6	0.11	6.3	4.45	0.010	6.6

Moving on to the films and 10-PPy6, as can be seen from the inset of Figure 6 and Table 5, the *M*_s_ varies significantly despite all three samples being composed of the same batch. The highest *M*_*s*_ is reported for the A-Film-10-PPy6, which was cured in the presence of a magnetic field. Generally, PEO-Fe_3_O_4_ systems are prone to spin canting effects,^55,56^ which, in our case, are likely present due to the variations in the exchange bias field (*H*_EA_), which is not present for either of the PPy nanotubes, and the slight variations of the intrinsic coercive field (*H*_ci_) and mass-specific susceptibility (χ_m_), as seen in Table 5. Differences in *H*_EA_, however, were reported due to distinctions in particle structuring within MREs without changes in *M*_s_.^57^ The dipolar interactions within the samples are also likely present, given the differences in remanence (*M*_r_).^58^ The above results suggest that, apart from the synthesis conditions of the PPy tubes, the preparation of films can affect the surface interactions between the magnetite and the other components^59^ which can be very important for potential SSEs or separators. There can be many origins of the differences in the magnetic properties of such composites;^60^ all of them are rather complex, which is out of the scope of this work. However, given the results, it should be considered an appropriate topic for future studies.

### Rheology

Frequency sweeps were performed at room temperature, with selected results presented in Figure 7a. All samples exhibit solid-like behavior (including those not shown), with both moduli being frequency-independent. Both moduli, and especially *G″,* are fluctuating in a few areas, which is expected due to the high stiffness of the samples that promotes slip. The average value of *G′* from the experimental window is shown as *G′*_P_ in Figure 7b and demonstrates values over 10 MPa, which should be sufficient for prolonged battery use without dendrite formation. For the PEO-M sample, Na-CMC acts as a plasticizer, lowering *G′*_P_. The presence of filler nanotubes increases both moduli, with *G′*_P_ being increased by almost an order of magnitude. Samples with 5% filler nanotubes showed a similar modulus to pure PEO/PEO-M. Only the 5-PPy6 sample overcame the plasticizing effect of Na-CMC, while the modulus of 5-PPy2.5 remained relatively the same as PEO-M. Doubling the nanotube’s mass fraction significantly increased *G′*_P_ which was expected for such composites^61,62^ and has been observed for nanotube-based systems.^63^ The increase is significantly higher for the 10-PPy2.5 sample, which shows the highest *G′*_P_ among all the tested samples.

**Figure 7:**
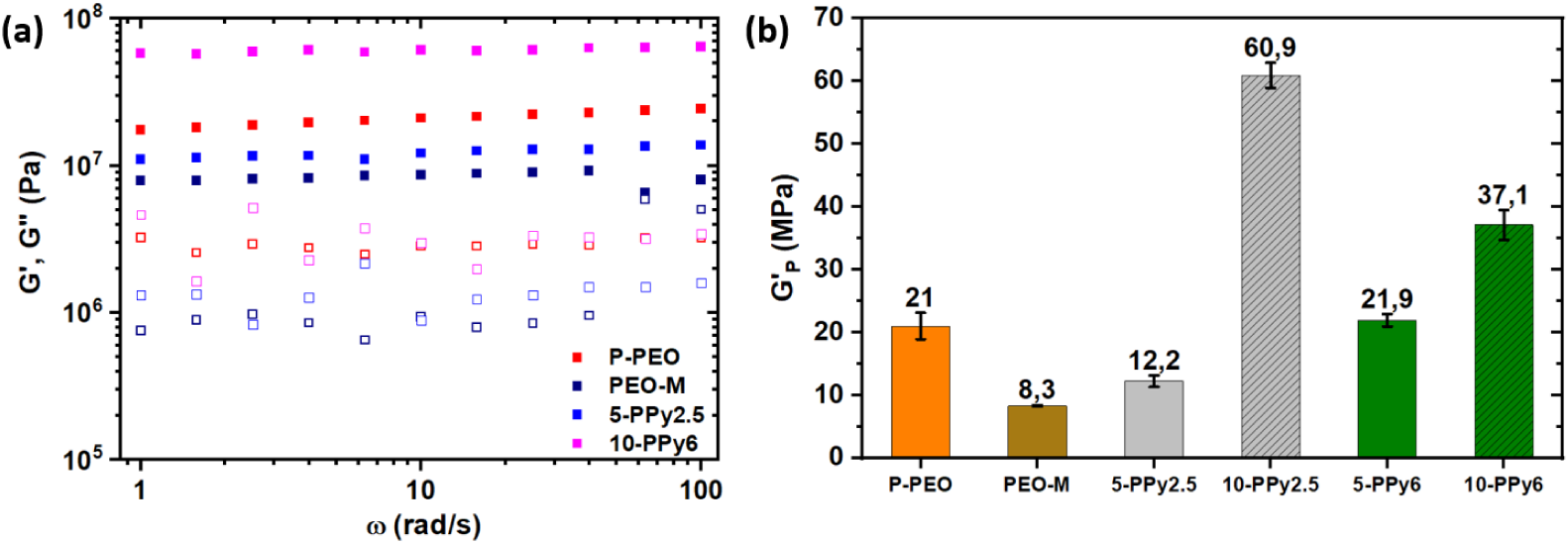
a) Frequency sweeps for P-PEO, PEO-M, and PEO/PPy2.5/PPy6-based SSEs at 25 °C; closed and open symbols correspond to *G*′ and *G*″, respectively. b) Average *G*′_P_ extracted from the corresponding frequency sweeps for all samples.

Given that batteries can operate at elevated temperatures, it is important to evaluate the performance of the pellets accordingly. Thus, the samples were also heated to 80 °C from room temperature, and once a steady state was reached, frequency sweeps were performed (Figure 8). Both moduli became frequency-dependent, which is typical for PEO-based composites at elevated temperatures. Regarding the values of the moduli, the same trend as for the samples at 25 °C (Figure 7b) was observed, with the 10-PPy2.5 sample showing the highest values. The PEO-M was the only exception, where both moduli were above the pure, likely due to interactions between PEO and Na-CMC around the melting temperature. For both pure and PEO-M samples, it is evident that in the low-frequency regime, a crossover between *G′* and *G″* occurs, with the samples becoming liquid-like. That was not the case for the filled MREs, which showed clear solid-like behavior, with *G′* being between 100 kPa and one MPa for the 10-PPy2.5 and 10-PPy6 specimens, respectively. As a result, pellets composed of the PPy filler preserved their ability to suppress tdendrite growth at elevated temperatures while not interacting with PEO at elevated temperatures. We achieve higher modulus values than cross-linked PEO^64^ and comparable mechanical properties to other PEO-based composites prepared specifically to inhibit dendrite growth.^21,65^ To conclude, through the amounts of Na-CMC and PPy nanotubes and their types used during fabrication, it is possible to tune both the electrical and the mechanical properties of the material, both being important for solid-state batteries.

**Figure 8:**
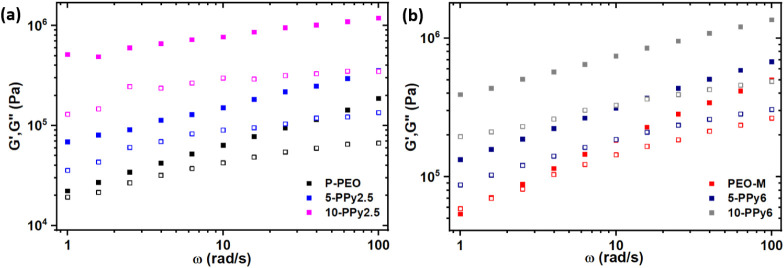
Frequency sweeps for (a) P-PEO and PEO/PPy2.5-based, (b) PEO-M and PPy6- based SSEs at 80 °C; closed and open symbols correspond to *G*′ and *G*″, respectively.

## Conclusions

Our case study shows that incorporating magnetite-decorated PPy nanotubes into PEO/Na-CMC matrices reduces crystallinity and significantly enhances ionic conductivity. Improved synthesis yields uniformly dispersed PPy/Fe_3_O_4_ nanotubes, with higher loading (*n* = 6) producing stronger integration into the polymer and confirmed magnetite formation by XRD. On the contrary, anisotropic structures did not significantly affect the conductivity of the composites. These structural features lead to conductivity values reaching ∼10^–4^ S/cm, especially in pelletized samples where compaction promotes efficient ion transport.

PPy_6_ composites also retain the magnetic behavior characteristic of iron oxide nanoparticles, and their magnetic response depends strongly on the preparation method. Mechanically, the materials exhibit shear moduli above 10 MPa and maintain solid-like stability at 80 °C, which is advantageous for suppressing dendrite growth.

Overall, the synergistic combination of PEO, PPy nanotubes, and magnetite nanoparticles produces structurally organized, mechanically robust composites with enhanced ionic transport, highlighting their potential as solid polymer electrolytes for next-generation electrochemical devices.
